# Racial/Ethnic Disparities in Mutual Help Group Participation for Substance Use Problems

**DOI:** 10.35946/arcr.v41.1.03

**Published:** 2021-03-11

**Authors:** Sarah E. Zemore, Paul A. Gilbert, Miguel Pinedo, Shiori Tsutsumi, Briana McGeough, Daniel L. Dickerson

**Affiliations:** 1 Alcohol Research Group, Emeryville, California; 2 Department of Community and Behavioral Health, University of Iowa, Iowa City, Iowa; 3 Center for Health and Social Policy, College of Education-Kinesiology and Health Education, University of Texas, Austin, Texas; 4 School of Environment and Society, Department of Social and Human Sciences, Tokyo Institute of Technology, Tokyo, Japan; 5 Cofrin Logan Center for Addiction Research and Treatment, School of Social Welfare, University of Kansas, Lawrence, Kansas; 6 Integrated Substance Abuse Programs, University of California, Los Angeles, California

**Keywords:** race/ethnicity, African American, Hispanic, Native American, mutual help, self-help, alcohol

## Abstract

Mutual help groups are a ubiquitous component of the substance abuse treatment system in the United States, showing demonstrated effectiveness as a treatment adjunct; so, it is paramount to understand whether they are as appealing to, and as effective for, racial or ethnic minority groups as they are for Whites. Nonetheless, no known comprehensive reviews have examined whether there are racial/ethnic disparities in mutual help group participation. Accordingly, this study comprehensively reviewed the U.S. literature on racial/ethnic disparities in mutual help participation among adults and adolescents with substance use disorder treatment need. The study identified 19 articles comparing mutual help participation across specific racial/ethnic minority groups and Whites, including eight national epidemiological studies and 11 treatment/community studies. Most compared Latinx and/or Black adults to White adults, and all but two analyzed 12-step participation, with others examining “self-help” attendance. Across studies, racial/ethnic comparisons yielded mostly null (*N* = 17) and mixed (*N* = 9) effects, though some findings were consistent with a racial/ethnic disparity (*N* = 6) or minority advantage (*N* = 3). Findings were weakly suggestive of disparities for Latinx populations (especially immigrants, women, and adolescents) as well as for Black women and adolescents. Overall, data were sparse, inconsistent, and dated, highlighting the need for additional studies in this area.

## INTRODUCTION

Racial/ethnic minority groups comprise a large proportion of the U.S. population and evidence a substantial need for treatment of substance use disorder (SUD). Analysis of the most recent, reliable data available—the 2018 National Survey on Drug Use and Health (NSDUH)^1^—found that the prevalence of past-year SUD among those age 12 and older was higher among some racial/ethnic minority groups than Whites. Compared to Whites (with a prevalence rate of 7.7%), the prevalence of past-year SUD was 31% higher among American Indians or Alaska Natives (10.1%), 21% higher among Native Hawaiians or Other Pacific Islanders (9.3%), and 16% higher among multiracial U.S. residents (8.9%). The prevalence rate for Whites was similar to those for Hispanic or Latino populations (7.1%) and Black or African American populations (6.9%). Prevalence among Asians was low overall (4.8%), though other studies suggest that substance use problems may be elevated in some ethnic subgroups (e.g., Koreans) and in Asian American young adults.^2^–^4^ Parallel patterns emerged for alcohol and illicit drug use disorders, revealing elevated rates among American Indians or Alaska Natives, Native Hawaiians or Other Pacific Islanders, and multiracial respondents in both cases.

Participation in mutual help groups (also known as self-help groups), including 12-step groups such as Alcoholics Anonymous (AA), is an integral and nearly ubiquitous component of the U.S. SUD treatment system^5^–^7^ and a typical constituent of mandated treatment.^8^ Moreover, 12-step participation—in conjunction with specialty SUD treatment (i.e., formal SUD treatment, such as that delivered in outpatient or residential treatment programs)—is also highly effective in treating SUD for typical treatment populations overall.^9^–^14^ Indeed, 12-step facilitation (TSF) interventions, which are designed to enhance involvement by (for example) explaining 12-step principles and culture, have repeatedly, if not universally, achieved better substance use outcomes than both usual treatment alone and gold standard treatments, such as cognitive behavioral therapy.^15^ Emerging studies also have examined, and found support for, the effectiveness of abstinence-based, secular mutual help alternatives to the 12-step approach.^16^–^18^ For instance, one recent study compared the effectiveness of 12-step groups and several abstinence-based alternatives—namely, Women for Sobriety, Self-Management and Recovery Training (SMART Recovery), and LifeRing Secular Recovery (LifeRing)—among current attendees with alcohol use disorder (AUD) over 1 year. Results indicated equally strong relationships between higher involvement and better substance use outcomes regardless of mutual help group choice and, unexpectedly, higher group cohesion and satisfaction in Women for Sobriety, SMART Recovery, and LifeRing versus 12-step groups.^17^,^18^

Together, the prevalence and effectiveness of mutual help groups highlight a critical need to understand the nature and extent of racial/ethnic disparities in mutual help group participation for substance use problems. Given that mutual help groups are a key resource for supporting recovery, any racial/ethnic disparity in mutual help participation connotes a potential health disadvantage for racial/ethnic minority groups that is worthy of investigation.^19^ Investigation of disparities in mutual help group participation is particularly valuable because there are reasons to believe that racial/ethnic minority groups (and especially immigrants) experience unique barriers to mutual help participation (e.g., racial/ethnic discrimination) as well as more barriers to help-seeking generally, described below. Accordingly, the present study offers a comprehensive review of empirical research on racial/ethnic disparities in mutual help group participation, addressing research on individuals with alcohol and/or drug problems. Although others have summarized the literature on racial/ethnic disparities related to mutual help groups,^10^,^20^,^21^ this study is the first known comprehensive review. Attention is focused predominantly on racial/ethnic disparities related to 12-step groups (and especially AA) because these groups have been the dominant focus of existing literature; however, the review also discusses alternatives to 12-step groups. Results will inform attempts to maximize SUD treatment effectiveness among racial/ethnic minority groups as well as future research aiming to understand recovery and pathways for recovery among racial/ethnic minority populations.

## UNIQUE BARRIERS TO PARTICIPATION IN MUTUAL HELP GROUPS

Several qualitative studies on the experiences of racial/ethnic minority individuals in 12-step groups/AA have concluded that these individuals may face unique barriers to full mutual help group participation and benefit. For example, Jilek-Aall suggested that AA can be off-putting to American Indians because attending AA may be equated with rejection of one’s Indian identity and culture; because AA’s worldview and practices (e.g., focus on confession-like speeches and Christian religiosity) are not consistent with those of American Indians; and because of miscommunication, barriers to trust, and discrimination by Whites.^22^,^23^ Venner and colleagues’ more recent, qualitative study likewise concluded that American Indians may avoid AA because they see it as “for White men,” because aspects of the program are not consistent with their beliefs and preferences, and because they feel scrutinized in AA.^24^ For some of the same reasons, others have argued that mainstream AA can be a poor fit for Black^25^–^27^ and Latinx^28^ people with substance use problems.

This literature broadly illuminates three distinct mechanisms that may create discomfort for racial/ethnic minority individuals in the context of mutual help groups. Racial/ethnic minority individuals may (a) perceive that their people and culture are not well represented within a given mutual help group’s founding, history, membership, and/or leadership, generating concern and mistrust; (b) perceive that a given mutual help group’s philosophy, values, and practices run counter to those of their own culture; and (c) experience challenging, current social contexts within a given mutual help group, such as heightened scrutiny, prejudice, and discrimination. These barriers could influence racial/ethnic minority individuals to avoid meetings and/or to participate in circumscribed ways that limit the benefits of participation, such as avoiding talking, avoiding sensitive disclosures, and failing to seek a 12-step sponsor. Although not a focus of the above studies, language barriers also could diminish or preclude participation for racial/ethnic minority groups, especially recent immigrants and those with low acculturation to U.S. society.

Counter to these arguments, some evidence suggests that such differences can be at least partially overcome. In principle, 12-step groups are open to adaptation,^29^,^30^ and they have proliferated (in sometimes adapted form) in many countries throughout the world, suggesting the potential for wide if not universal appeal.^31^ Furthermore, 12-step groups have been culturally adapted specifically for American Indian and Alaska Native,^22^,^23^,^32^,^33^ Black,^26^,^27^ and Latinx^28^,^34^,^35^ populations. For American Indians and Alaska Natives, the Medicine Wheel and 12 Steps program blends Native American traditional teachings with the 12 Steps of Alcoholics Anonymous to provide culture-specific recovery assistance for Native Americans.^32^ In this program, each step may be worded differently from its AA wording, and the steps are presented in a circle rather than as a straight-line listing to ensure cultural appropriateness. Also, this program states that being “in recovery” requires a further journey to wellness by going beyond “clean and sober,” by pursuing a journey of healing and balance—mentally, physically, emotionally, and spiritually. This highlights that racial/ethnic minority individuals may have distinct concepts of recovery that should (and can) be addressed in cultural adaption.

Nonetheless, appropriately adapted meetings may not be available and accessible to all racial/ethnic minority groups and subgroups. For example, Asian Americans may face especially serious barriers to 12-step participation given the prohibitions common to many Asian cultures against publicly acknowledging addiction^36^,^37^ and given the heterogeneous composition and small number of Asian Americans in the United States, which may inhibit the growth of culturally adapted meetings. Racial/ethnic minority individuals living outside of major metropolitan areas or ethnic enclaves also may be at a disadvantage, due to their restricted access to culturally adapted meetings;^29^ and recent immigrants and others low on acculturation may struggle with cultural mismatch regardless of the availability of culturally adapted meetings, as adapted meetings in the United States still may fail to adequately reflect their cultures of origin.^28^

## GENERAL BARRIERS TO HELP SEEKING

Quantitative and qualitative studies also suggest that racial/ethnic minority groups face greater barriers to seeking help for SUD more generally, which likewise could influence mutual help group participation and benefits. Multiple studies conducted with U.S. national samples have reported lower rates of specialty SUD treatment utilization among Latinx (vs. White) individuals with SUD,^38^–^44^ with studies suggesting particularly limited utilization among foreign-born and Spanish-speaking Latinx subgroups.^45^–^47^ National studies in the United States also have reported disparities in specialty SUD treatment utilization among Asian Americans (vs. Whites)^4^,^48^ and lower SUD treatment retention among both Black and Latinx (vs. White) individuals.^49^,^50^ These studies provide compelling evidence of racial/ethnic disparities in treatment utilization and retention because they used nationally representative samples, restricted analysis to those with an SUD, and often controlled for problem severity.

A parallel evidence base has addressed general barriers to seeking help for an SUD, focusing mostly on Latinx and Black populations.^42^,^47^,^51^–^55^ Studies (most addressing multiple barriers simultaneously) have described increased barriers facing Latinx and Black populations in several categories, including logistic barriers (e.g., difficulties with finding treatment, paying/qualifying for treatment, obtaining transportation, handling family and work responsibilities), attitudinal barriers (e.g., lack of perceived treatment need, lack of perceived treatment effectiveness), social and legal barriers (e.g., lack of social support/pressure for treatment seeking, stigma, concerns about deportation, concerns about retaining child custody), and cultural barriers (e.g., lack of culturally adapted treatments, lack of racial/ethnic minority group representation among clients and staff).

Although parallel studies have not been conducted to explore barriers to mutual help group participation per se, many of the above barriers could plausibly affect mutual help group participation. Logistic barriers may be especially salient for recent immigrants and economically disadvantaged groups. For example, recent immigrants and impoverished members of racial/ethnic minority groups may face particular challenges in locating appropriate meetings, obtaining transportation to meetings, and handling competing responsibilities. That said, impacts of certain logistic and legal barriers to help seeking in general terms may be somewhat mitigated when considering mutual help group participation specifically. This is because 12-step meetings are widely available (i.e., located in accessible community settings), free, and independent of governmental institutions.

A last point worthy of attention is that disparities in treatment utilization and retention among Latinx, Black, and Asian populations may themselves constitute barriers to mutual help group participation among affected groups because specialty treatment constitutes a major route to mutual help group involvement (and especially 12-step involvement). Referral to meetings by treatment staff is perhaps the predominant route to 12-step participation, so those who do not attend (or attend less) treatment may be less likely to participate in 12-step groups. Toward this point, 32% of respondents to the 2014 AA Membership Survey reported direct referral from a treatment facility, and 59% reported receiving some treatment/counseling related to their drinking before coming to AA; among the latter, 74% said this experience played an important part in directing them to AA.^8^ Referral to 12-step by medical and mental health professionals is also common,^8^ which may similarly disadvantage Latinx and Black individuals because they are less likely than Whites to regularly access primary care and mental health care.^56^–^59^

The discussion above paints a complex picture of the potential for racial/ethnic disparities related to mutual help groups. It suggests that, although any racial/ethnic minority individual could experience multiple barriers to mutual help group participation, mitigating factors may alter the impacts of these barriers. In lieu of study hypotheses, this review therefore offers two questions:

1. What is the extent and nature of quantitative research on racial/ethnic disparities in mutual help group participation?
2. Do existing studies suggest racial/ethnic disparities in mutual help group participation, and for whom?

In addressing the second question, the review initially examines national studies and treatment/community studies separately, given their differences in rigor and sampling strategies. However, in view of the limited evidence base, results from both study types are synthesized to formulate overarching conclusions.

## METHODS

### Approach and Search Strategy

The current review employed a narrative review strategy strengthened by incorporation of key aspects of systematic reviews, including systematic search procedures and study coding. To locate relevant publications, PubMed and PsycINFO were searched using the following search terms and combinations thereof: mutual help, self-help, mutual aid, Alcoholics Anonymous, Narcotics Anonymous, Cocaine Anonymous, Marijuana Anonymous, 12-step, twelve-step, SMART Recovery, LifeRing, Women for Sobriety, alcohol, substance, drug, Black, African American, Latino, Hispanic, Asian American, American Indian, Native American, Alaska Native, race, and ethnicity. Reference lists of relevant articles and related-citation links also were examined.

### Focal Variables and Study Inclusion and Exclusion Criteria

This review examined associations between racial/ethnic self-identification (the independent variable) and mutual help participation (the outcome), defined as meeting attendance and/or participation in key activities. The review included only original, quantitative articles describing the results of U.S. studies; published in English-language, peer-reviewed journals; and analyzing the presence or extent of mutual help participation across two or more specific racial/ethnic groups with SUD treatment need—as indicated by the presence of an alcohol problem and/or drug use/a drug problem. The review included studies on both adults and adolescents, using no publication date restrictions. Studies were excluded from review if they (1) analyzed only one racial/ethnic group; (2) compared Whites to a combined sample of racial/ethnic minority groups; (3) omitted statistical tests of racial/ethnic differences in mutual help group participation or data sufficient for such tests; or (4) presented results for subsamples of racial/ethnic minority groups where data for the larger racial/ethnic populations were published elsewhere.

### Analysis and Summary of Findings

Where statistical comparisons were not provided, this review’s lead author conducted bivariate comparisons (i.e., Pearson chi-square tests) using raw, published data. Study characteristics and relevant results were summarized in two descriptive tables. A third table was used to summarize the main results for each racial/ethnic subgroup separately. This table coded results for racial/ethnic comparisons across all mutual help participation outcomes for a given study, but relative only to a specific racial/ethnic group (e.g., coding results for Latinx-White comparisons on all study measures of mutual help group participation at all time points). Results were coded as null, mixed, entirely consistent with lower minority-group participation (a disparity), or entirely consistent with higher minority-group participation (a minority advantage); results were coded as “mixed” when they differed across outcomes, data sources, and/or subgroups (e.g., genders). Marginally significant results (i.e., .05 < *p* < .10) were coded as significant, not null, for this purpose.

## RESULTS

### National, Epidemiological, Cross-Sectional Studies

Table 1 presents the characteristics and key results of identified national epidemiological studies examining racial/ethnic differences in mutual help group participation; all were cross-sectional (*N* = 8 studies).^38^–^42^,^60^–^62^ Data sources were the 1995–2010 National Alcohol Survey (NAS) series, the 1991–1992 National Longitudinal Alcohol Epidemiologic Survey (NLAES), the 2001–2002 and 2004–2005 National Epidemiologic Surveys on Alcohol and Related Conditions (NESARC), and the 2001–2013 NSDUH series, yielding six unique data sets. No studies addressed adults over the past decade. As shown in Table 1, key racial/ethnic subgroups were relatively large (all *N* > 100), excepting those for Asian American/Native Hawaiian/Pacific Islander (*N*= 99) and Native Hawaiian/Pacific Islander (*N*= 68) groups. All but two studies targeted Latinx and/or Black populations, and only one targeted adolescents. All but two studies^40^,^42^ aggregated across nativity and gender when examining racial/ethnic differences. However, all studies including Latinx respondents, excepting the NLAES, reported providing Spanish-language interviews, allowing participation of those not fluent in English. Half targeted those with AUD only, with the others targeting other drug use disorders also or exclusively. All eight studies analyzed AA/12-step or “self-help” attendance and were limited to a measure of any versus no attendance, most using a lifetime time frame. Five conducted multivariate analyses.

Results were quite mixed, with three studies providing at least some evidence of disparities (i.e., Cummings et al., 2011;^39^ Mancini et al., 2015;^40^ Zemore et al., 2014^42^); three showing at least some evidence of a minority advantage (i.e., Chartier et al., 2011;^38^ Perron et al., 2009;^61^ Wu et al., 2016^62^); and two reporting entirely null results (i.e., Schmidt et al., 2007;^41^ Kaskutas et al., 2008^60^) for racial/ethnic differences in mutual help group participation. (See also Table 3.)

### Treatment and Community Studies

Table 2 presents the characteristics and key results of identified treatment- and community-based studies examining racial/ethnic differences in mutual help group participation (*N* = 11 studies).^29^,^63^–^72^ Studies represent 10 unique data sources, many dated—especially for Latinx-White and Black-White comparisons. Seven of the 11 reported total *samples* of less than 100 for key racial/ethnic subgroups. All but two studies targeted Latinx and/or Black populations exclusively, and all but one targeted adults. All 11 studies aggregated across nativity and gender groups for analysis, and no studies sampling Latinx respondents reported the use of Spanish-language interviews. Five targeted individuals seeking alcohol-related services (the remainder studying populations seeking SUD services), and all studied AA/12-step participation. Contrasting with the epidemiological studies, most (six) captured level of (vs. any/no) participation, at least in addition to any/no participation, and several examined activity participation as well as attendance at meetings. Most (eight) conducted only bivariate analyses or analyses controlling for treatment condition or time alone.

Results were again mixed, with three studies providing at least some evidence of disparities (i.e., Arroyo et al., 1998;^65^ Tonigan et al., 1998;^66^ Tonigan, 2003^69^); three showing at least some evidence of one or more minority advantages (i.e., Humphreys et al., 1991;^63^ Kingree et al., 1997;^64^ Tonigan et al., 2013^72^), one reporting countervailing results (i.e., Kaskutas et al., 1999^67^), and four reporting entirely null results (i.e., Humphreys and Woods, 1993;^29^ Hillhouse and Fiorentine, 2001;^68^ Goebert and Nishimura, 2011;^70^ Krentzman et al., 2012^71^). (See also Table 3.)

### Overall Summary of Results

Table 3 summarizes the findings of Tables 1 and 2 separately for comparisons involving Latinx; Black; American Indian or Alaska Native; Asian American, Native Hawaiian, or Other Pacific Islander; and multiracial respondents. As noted in the Methods, this summary table simultaneously codes results for comparisons across all mutual help participation outcomes for a given study, but relative only to a specific racial/ethnic group. This table reveals a lack of strong support for broad racial/ethnic differences in mutual help group participation. Of 35 comparisons between specific racial/ethnic minority groups and Whites on measures of mutual help group participation in a given study, nearly half (*N* = 17) yielded null results; only six comparisons yielded unequivocal support for racial/ethnic disparities, whereas nine yielded mixed results and three yielded unequivocal support for a minority advantage in mutual help group participation.

Nonetheless, it may be possible that results signify disparities for particular Latinx subgroups, as no results indicated a Latinx-White minority advantage and four results indicated Latinx-White disparities. Also, two of the three results coded as “mixed” reveal some disparities: Mancini et al. (2015) reported disparities in lifetime 12-step attendance among immigrant (but not U.S.-born) Latinx adults with lifetime drug use in a national sample,^40^ and Tonigan et al. (1998) reported disparities in AA attendance at the 12-month follow-up exclusively among Latinx adults with AUD in Project MATCH (with Latinx-White differences being nonsignificant at prior follow-ups).^66^ Black-White comparisons seem more consistent with null effects, with exceptions, as they yielded a range of results including many null results and several results suggesting a minority advantage. Data were very sparse for other racial/ethnic groups, with no evidence of disparities emerging.

## DISCUSSION

### Question 1: Extent and Type of Research on Disparities

The present review identified 19 studies addressing racial/ethnic disparities in mutual help group participation among those with SUD treatment need. This set includes eight national, epidemiological, cross-sectional studies that were generally well powered, incorporated Spanish-language interviews (allowing inclusion of Spanish speakers), and incorporated multivariate analyses with some adjustment for potential confounds. Also in this set were 11 treatment/community studies, strengths of which included consideration of level of mutual help group participation, as well as any or none, and analysis of multiple outcomes (including participation over time). Almost all studies used strong measures of SUD treatment need (i.e., SUD/AUD status), and rigorously conducted studies were included among both types.

Despite some strengths, the reviewed studies evidenced multiple design limitations, as follows.

- **Studies were generally dated and not optimally designed to assess racial/ethnic differences, with many studies showing inadequate power.** All but four studies analyzed data collected partially or entirely more than a decade ago. U.S. demographics are in constant flux—for example, recent years have witnessed rapid growth of racial/ethnic minority populations and shifts in Latinx settlement patterns^73^,^74^—so older findings may not represent current conditions in the United States. Existing analyses also seemed to be largely secondary analyses, and most treatment/community studies were underpowered for detecting differences in mutual help group participation across racial/ethnic groups. Even assuming bivariate analysis and a continuous outcome, tests require at least 99 participants per group to detect a small-to-medium effect size (Cohen’s *d* = .40) with adequate power (β = .80);^75^ power is even more limited given multivariate analysis and a dichotomous outcome.
- **Studies provided limited data on racial/ethnic minority groups other than Latinx and Black populations, and on important racial/ethnic subgroups including immigrants, women, and adolescents.** Identified studies included just two or three studies each on American Indian or Alaska Native, Asian American, and Native Hawaiian and Other Pacific Islander populations. One study examined immigrants (Mancini et al., 2015),^40^ one study examined women separately (Zemore et al., 2014),^42^ and two studies examined adolescents (Cummings et al., 2011;^39^ Krentzman et al., 2012^71^). Yet, all of the studies focusing on immigrants, women, and adolescents reported disparities, underlining the importance of studying these populations.
- **Regardless of racial/ethnic group focus, treatment/community studies sampled a restricted range of populations, further limiting generalizability.** Although most national studies provided Spanish-language interviews, none of the treatment/community studies did so. Hence, these studies presumably excluded all those not fluent in English, who differ widely from English speakers on substance use and help-seeking patterns.^58^–^60^,^76^ Treatment/community studies also focused on a small set of predominantly urban samples. This is an important limitation because, as discussed, geography may moderate racial/ethnic disparities in mutual help group participation and benefits, with those living outside of ethnic enclaves likely to show increased disparities.
- **Studies focused predominantly on respondents with AUD, and all studies examined AA/ 12-step participation or global “self-help” participation.** Very few studies focused on populations with a drug use disorder (DUD), and none examined 12-step alternatives such as SMART Recovery, a rapidly growing recovery resource. Consequently, findings cannot be confidently generalized to populations with DUD—comprising large proportions of those with SUD treatment need^77^,^78^—or to 12-step alternatives.

Studies also showed limitations associated with their measures and analysis.

- **Studies often relied on crude, dichotomous measures of 12-step participation (especially in national samples).** Most problematic, national studies relied completely on any/no (usually lifetime) measures of mutual help participation. Although power considerations may preclude use of more detailed measures, this means that national data cannot speak to potential disparities in involvement patterns, such as a tendency for Latinx people to discontinue 12-step involvement more frequently than Whites. Most studies also neglected to measure activity participation, though much of the effectiveness of 12-step participation can be attributed to activity involvement, such as obtaining a sponsor.^79^
- **Studies relied quite heavily on bivariate analyses, and they neglected potential confounds.** Even where multivariate analyses were conducted, very few controlled for differences in SUD severity. Neglect of SUD severity is particularly concerning: Where SUD severity is not controlled, any findings may be distorted by an association between race/ethnicity and problem severity, as higher SUD severity has been consistently associated with greater 12-step participation^80^ ^83^ (and indeed implies greater treatment need). These limitations should be addressed in future research.

### Question 2: Findings for Racial/Ethnic Disparities

As a whole, studies did not provide strong evidence of racial/ethnic disparities for any racial/ethnic group. Still, six studies revealed some evidence of Latinx-White disparities in mutual help group participation, including national, epidemiological studies using NSDUH, NESARC, and NAS data (Cummings et al., 2011;^39^ Mancini et al., 2015;^40^ Zemore et al., 2014^42^) and treatment/community studies analyzing data from a New Mexico outpatient SUD treatment program and Project MATCH (Arroyo et al., 1998;^65^ Tonigan et al., 1998;^66^ Tonigan et al., 2003^69^). Results of a NESARC analysis by Mancini et al. (2015) are particularly notable, showing a sizeable disparity among Latinx immigrants (vs. Whites) reporting drug use across bivariate and multivariate analyses; analyses revealed significantly lower odds of lifetime 12-step attendance among Latinx immigrants vs. Whites (multivariate *OR* = 0.39)*.*^40^ Results call for cautious interpretation because, in addition to targeting any/no participation, analyses considered all those with any drug use and did not control for drug use severity. Still, similar results emerged in a within-group (noncomparative) study of Latinx respondents with lifetime AUD interviewed for the 2000–2010 NAS,^60^ which reported significantly greater lifetime 12-step attendance among those interviewed in English vs. Spanish (multivariate *OR* = 3.20) despite comprehensively controlling for severity. As this review’s Introduction suggests, multiple studies^58^–^60^ likewise have found diminished use of specialty treatment (and AUD services broadly) among Latinx immigrants and those speaking predominantly Spanish. In general, Latinx immigrants may tend to use fewer services, including mutual help groups, and/or prefer services not fully captured in the literature, such as services in their countries of origin and/or nontraditional services in the United States. For example, literature has documented some use among Latinx populations of *anexos,* which are community-based recovery homes that draw on AA principles and provide care to primarily male Latinx migrants and immigrants.^84^,^85^ Regardless, these disparities raise questions as to whether existing recovery-related services are sufficient to support recovery for Latinx populations.

Also notable, studies reported substantial Latinx-White disparities in analyses targeting women (Zemore et al., 2014)^42^ and adolescents (Cummings et al., 2011),^39^ again across bivariate and multivariate analyses. These studies are notable because they analyzed large, national data sets and employed multivariate analyses. Moreover, the pattern of effects in each was similar across multiple outcomes, and results were not undermined by findings for null or contrary results in other studies. Using NAS data, Zemore et al. (2014) reported significantly lower odds of lifetime 12-step attendance among Latinx versus White women with lifetime AUD (multivariate *OR,* Model 3 = 0.30).^42^ Findings also revealed large disparities in 12-step attendance among Latinx versus White men and Black versus White women, along with the same pattern of disparities for specialty treatment, perhaps implying general obstacles to help seeking among all Latinx individuals and Black women. Using NSDUH data, Cummings et al. (2011) reported substantially lower rates of 12-step attendance among both Latinx and Black (vs. White) adolescents, again in both bivariate and multivariate models; they also found the same pattern of disparities for any SUD treatment and treatment in medical settings.^39^ Cummings et al. speculated that these disparities may be explained by lack of SUD services in Latinx and Black neighborhoods; low acculturation among Latinx adolescents; and racial/ethnic differences in stigma, attitudes, and cultural beliefs concerning behavioral health problems and treatment.^39^ It is also possible that there are detrimental, cumulative effects of being both young and belonging to a racial/ethnic minority group, such as intensified stigma and difficulties with “fitting in” in treatment and mutual help group settings.

Otherwise, findings for Latinx-White disparities in the general population and among treatment/community samples were quite mixed. Existing data are not sufficient to confidently establish those factors driving variation in results across studies, but variation across national epidemiological studies may at least partially reflect differences in how studies obtained respondents from racial/ethnic minority groups. For example, at the time data relevant to this review were collected, the NSDUH did not oversample racial/ethnic minority groups; the NESARC oversampled racial/ethnic minority groups, although information on oversampling methods could not be located; and the NAS targeted high-minority-density areas. The NAS approach apparently yielded the strongest representation of Latinx respondents low on acculturation, with 45% of Latinx respondents interviewed in Spanish across the pooled 1995–2005 NAS^60^ (vs. 16% in the 2001–2002 NESARC^86^ and a weighted 23% in the 2001–2013 NSDUH^87^). If disparities are strongest for Latinx populations low on acculturation, as seems evident, this may explain why Zemore et al. (2014) reported Latinx-White disparities for both men and women,^42^ and other national studies did not.

Meanwhile, respondents’ geographic context—and specifically, access to racial/ethnic minority–inclusive and culturally adapted meetings in the community—may have contributed to variation in results for the treatment/community studies. Humphreys and Woods (1993) have argued that geography and race/ethnicity interact to affect mutual help group participation, and specifically that people with SUD may prefer to attend meetings in areas where their own race/ethnicity is well represented.^29^ In fact, their study of treatment seekers with SUD found that Black participants were more likely to attend a mutual-help group if they resided in a predominantly Black area; similarly, White participants were more likely to attend a mutual help group if they resided in a predominantly White area. Accordingly, the inconsistent results for treatment/community studies may reflect differences in the samples’ access to minority-inclusive and culturally adapted meetings. This seems a plausible explanation for the null findings reported for Latinx-White differences in mutual help group participation in the diverse Los Angeles metropolitan area (i.e., Hillhouse & Fiorentine, 2001),^68^ versus other studies reporting Latinx-White disparities with samples drawn from less metropolitan areas (i.e., the Arroyo^65^ and Tonigan^66^,^69^ studies). Future studies of racial/ethnic disparities that explicitly consider the acculturation status of respondents and access to minority-inclusive and culturally tailored meetings will be needed to better evaluate these possibilities.

Regarding Black populations, studies produced little evidence for disparities in mutual help group participation, and several studies reported evidence of greater mutual help group participation among Blacks than Whites (i.e., Perron et al., 2009;^61^ Humphreys et al., 1991;^63^ Kingree et al., 1997;^64^ Kaskutas et al., 1999^67^). (Exceptions are the notable studies targeting women and adolescents described above.) Several factors could explain the relatively strong participation rates among Black people with SUD treatment need overall. As noted above, studies generally did not control for SUD severity, so they may have missed disparities that would arise when accounting for intensity of treatment need. Another possibility is that prevalent religiosity/spirituality among Black populations^88^,^89^ may make 12-step groups particularly appealing, counteracting any obstacles to participation. Other explanatory factors may include the higher rate of SUD treatment coercion among Black versus White populations,^90^ which can include coercion to 12-step group participation, and differences in program emphasis on 12-step principles and participation within programs serving predominantly Blacks vs. Whites.^29^ The mixed findings for Black-White differences may reflect chance, geographic factors, and sample characteristics (e.g., proportion with DUD, as those with DUD may be more likely than those with AUD to experience coercion). Findings from the few studies of American Indian, Alaska Native, Asian American, Native Hawaiian, and Other Pacific Islander populations provided no indication of disparities, but the sparse data preclude strong conclusions.

### Future Research Needs and Clinical Implications

The sparse and inconsistent evidence base described above highlights a need for additional research on racial/ethnic disparities in mutual help group participation. In particular, current epidemiological studies are needed to better investigate potential disparities, ideally using sophisticated measures of mutual help involvement and accounting for potential differences in clinical severity. NSDUH data would be especially well suited for examination of current disparities in rates of mutual help group participation. Well-powered treatment/community studies are also important to address the potential for racial/ethnic disparities in mutual help group involvement patterns over time, including involvement in key activities such as sponsoring relationships. Both epidemiological and treatment/community studies should pay particular heed to individual and contextual factors—such as gender, age, acculturation level, and access to minority-inclusive and culturally tailored meetings—that may affect participation in mutual help groups. Meanwhile, qualitative studies would be useful to capture the self-perceived needs and barriers of racial/ethnic minorities regarding mutual help groups. Studies might focus particularly on Latinx, American Indian, Alaska Native, Asian American, Native Hawaiian, and other Pacific Islander populations as well as racial/ethnic minority immigrants, women, and adolescents.

Studies also might address a wider range of mutual help groups as recovery resources for racial/ethnic minority individuals, such as SMART Recovery. SMART is the largest known alternative to 12-step groups with more than 2,200 meetings in the United States. SMART’s philosophical focus on empowerment (vs. surrender) may be especially appealing and appropriate for racial/ethnic minority individuals, who are likely to face disenfranchisement by the majority culture. Similarly, research is needed to examine the use of online mutual help meetings and resources among racial/ethnic minority groups. Many mutual help options, including 12-step groups, have online meetings and forums,^17^,^91^ and aspects of these resources (e.g., their greater anonymity and ease of access) may be particularly appealing to racial/ethnic minority individuals. Importantly, online meetings have the potential for substantial cultural tailoring because they are geographically unlimited: A given meeting might be tailored to a very specific subgroup and draw attendees from around the globe. Online recovery resources have become an especially salient target for research in recent times because they offer ongoing, peer-based support during periods of social distancing.

Finally, studies are needed to address racial/ethnic disparities in the relationship between mutual help group participation and benefits. Few studies have addressed whether mutual help group participation is equally beneficial for racial/ethnic minority groups, with existing studies relying on a limited set of data sources.^65^,^69^,^72^,^92^,^93^ A key question is whether Spanish-language 12-step groups are effective among Spanish-speaking Latinx individuals, as 12-step participation may be a more accessible form of treatment than specialty care for disadvantaged Latinx populations, with Spanish meetings available in many urban centers (though the extent of foreign-language meetings in the United States has not been well documented).^94^,^95^ Broadly, it would be valuable to address the effectiveness of all prevalent mutual help group options and participation modes (i.e., in-person, online) for sustaining recovery among racial/ethnic minority individuals.

Together, the directions discussed above have the potential to advance the field not only by better describing existing disparities, but also by improving referral practices and interventions. Ultimately, studies might support the development and dissemination of new mutual help resources for racial/ethnic minority groups (e.g., culturally adapted meetings), which may be particularly important for those who underutilize specialty treatment and/or experience the heaviest burden of problems.

### Limitations of This Review

The current review may have omitted relevant studies because inclusion criteria were limited to published studies indexed in PubMed and PsycINFO. The review’s search strategy assumed that the vast majority of relevant studies would be indexed in these databases, but other databases may have yielded additional articles. Further, to be expeditious, this review drew upon, but did not fully adopt, guidelines from the PRISMA Group (Preferred Reporting Items for Systematic Reviews and Meta-Analyses).^96^ Future reviews may benefit from more formalized review procedures. Last, because the review was limited to U.S. studies, results cannot be generalized to other countries. (For international studies of AA, see Makela, 1996.^97^)

## FINAL CONCLUSIONS

Mutual help groups are a foundational and an effective component of the SUD treatment system in the United States, so it is critical to understand whether they are as appealing and effective for racial/ethnic minority groups as they are for Whites. Further, there are reasons to believe that racial/ethnic minorities (and especially immigrants) experience elevated barriers to participation in such groups, including barriers to mutual help group participation specifically and help seeking generally. Nonetheless, this comprehensive review found existing data to be insufficient to fully evaluate racial/ethnic disparities in mutual help group participation. Findings provided very tentative evidence for Latinx-White disparities, particularly among certain subgroups (i.e., immigrants, women, adolescents), as well as for disparities among Black women and adolescents. However, identified studies showed numerous limitations. Conclusions emphasize the need for additional research addressing the limitations of existing studies and targeting new and understudied questions, such as widening the lens to examine neglected mutual help group options and modes of participation.

## Figures and Tables

**Table 1:** National, Epidemiological, Cross-Sectional Studies of Racial/Ethnic Differences in Mutual Help Group Participation (*N* = 8)

Authors	Analytic Sample (All Mixed-Gender)	Data Source	Use of Spanish Interviews	Mutual Help Group Participation Outcome	Analysis	Results
Schmidt et al., 2007^41^	1,885 White, 704 Latinx, and 627 Black respondents	Adults with a lifetime AUD in the combined 1995 and 2000 NAS	Yes	AA attendance (yes vs. no) in one’s lifetime	Bivariate only	In the total sample, analyses showed no racial/ethnic differences.
Kaskutas et al., 2008^60^	1,029 White, 103 Latinx, 120 Black, and 73 Other respondents	Adults who attended a 12-step group in their lifetime (and prior to the past year) for an alcohol problem in the 2001–2002 NESARC	Yes	12-step attendance (yes vs. no) in the past year	Bivariate only	In the total sample, analyses showed no racial/ethnic differences.
Perron et al., 2009^61^	2,682 White, 595 Latinx, and 610 Black respondents	Adults with a lifetime DUD in the 2001–2002 NESARC	Yes	12-step attendance (yes vs. no) in one’s lifetime	Bivariate and multi-variate; controls were demographics and presence of other lifetime psychiatric disorders	Among those reporting any lifetime help seeking for a drug problem, both bivariate and multivariate analyses showed a significantly higher rate of 12-step attendance among Black vs. White respondents; Latinx and White respondents were equivalent. Among the total sample, bivariate analyses* similarly revealed a significantly higher rate of 12-step attendance among Black vs. White respondents.
Chartier et al., 2011^38^	For the NLAES, 6,016 White, 395 Latinx, and 598 Black respondents; for the NESARC, 8,011 White, 1,677 Latinx, and 1,579 Black respondents	Adults with a lifetime AUD in the 1991–1992 NLAES and the 2001–2002 NESARC	Yes for the NESARC, not stated for NLAES	12-step attendance (yes vs. no) in one’s lifetime	Bivariate and multivariate; controls were survey, demographics, insurance status, and alcohol severity	In the NLAES, bivariate analyses* showed no racial/ethnic differences. In NESARC, bivariate analyses* showed a significantly higher rate of 12-step attendance among Black vs. White respondents; Latinx and White respondents were equivalent. In pooled survey data, multivariate analyses showed a significantly higher rate of 12-step attendance among Latinx vs. White respondents; Black and White respondents were equivalent. A significant interaction indicated that the Latinx-White difference was diminished or reversed at higher levels of AUD severity.
Cummings et al., 2011^39^	8,506 White, 2,004 Latinx, 1,051 Black, 325 American Indian/Alaska Native, 181 Asian American, 68 Native Hawaiian/Pacific Islander, and 499 Multiracial respondents	Adolescents with past-year SUD in the combined 2001–2008 NSDUH	Yes	Self-help attendance (yes vs. no) in one’s lifetime	Bivariate and multivariate; controls were demographics, insurance status, any mental health treatment, type of SUD, and self-rated health	In the total sample, both bivariate and multivariate analyses showed significantly lower rates of self-help attendance among both Latinx and Black vs. White respondents; no other differences emerged.
Zemore et al., 2014^42^	3,788 White, 949 Latinx, and 738 Black respondents	Adults with lifetime AUD in combined 2000, 2005, and 2010 NAS	Yes	12-step attendance (yes vs. no) in one’s lifetime	Bivariate and multivariate; controls were survey, demographics, and dependence severity (as in Model 3)	Among men, both bivariate and multi-variate analyses showed a lower rate of 12-step attendance among Latinx vs. White respondents (though the difference was marginally significant in bivariate analyses); Black and White respondents were equivalent. Among women, both bivariate and multivariate analyses showed lower rates of 12-step attendance among Latinx and Black vs. White respondents.
Mancini et al., 2015^40^	5,754 White, 743 U.S.-born Latinx, and 280 Latinx immigrant respondents	Adults with lifetime drug use in the 2001–2002 and 2004–2005 NESARC (using variables from both)	Yes	12-step attendance (yes vs. no) in one’s lifetime	Bivariate and multivariate; controls were demographics, parental drug use history, and lifetime mood and anxiety disorders	In the total sample, both bivariate* and multivariate analyses showed a significantly lower rate of 12-step attendance among Latinx immigrant vs. White respondents; U.S.-born Latinx and White respondents were equivalent.
Wu et al., 2016^62^	4,361 White, 799 Hispanic, 459 Black, 141 American Indian/Alaska Native, 99 Native Hawaiian/Pacific-Islander/Asian American, 266 Multiracial respondents	Respondents age 12 and older reporting past-year OUD in the combined 2005–2013 NSDUH	Yes	Self-help attendance in the past year	Bivariate only	Among those reporting past-year use of any alcohol/drug services, analyses showed a significantly higher rate of self-help attendance among American Indian vs. White respondents; no other differences emerged.

*Note:* AA, Alcoholics Anonymous; AUD, alcohol use disorder; DUD, drug use disorder; NAS, National Alcohol Survey; NESARC, National Epidemiologic Survey on Alcohol and Related Conditions; NLAES, National Longitudinal Alcohol Epidemiologic Survey; NSDUH, National Survey on Drug Use and Health; OUD, opiate use disorder; SUD, substance use disorder.

* Analyses conducted on raw data by this review’s lead author.

**Table 2:** Treatment and Community Studies of Racial/Ethnic Differences in Mutual Help Group Participation (*N* = 11)

Authors	Analytic Sample (All Mixed-Gender)	Data Source and Analytic Design	Use of Spanish Interviews	Mutual Help Group Participation Outcome	Analysis	Results
Humphreys et al., 1991^63^	201 total with 115 Black respondents at follow-up; precise breakdown not provided	Adults with SUD recruited from 19 public SUD treatment programs (11 outpatient, 8 residential) in Michigan; longitudinal (follow-up rate 63%)	N/A	12-step attendance (any vs. no) between treatment end and 6-month follow-up	Bivariate only	In the total sample, analyses showed a significantly higher rate of 12-step attendance among Black vs. White respondents.
Humphreys & Woods, 1993^29^	267 White and 233 Black respondents at follow-up	Adult “substance abusers” (SUD status unclear) recruited from 22 SUD treatment programs in Michigan; longitudinal (follow-up rate 71%)	N/A	12-step attendance (any vs. no) in the prior 30 days at 12-month follow-up	Bivariate only	In the total sample, analyses* showed no racial/ethnic differences.
Kingree et al., 1997^64^	22 White and 78 Black respondents at follow-up	Adults with SUD recruited from a 120-day, 12-step–oriented addiction treatment program serving indigent poly-drug users, most with cocaine as drug of choice; longitudinal (follow-up rate 56%)	N/A	Scores on the AAAS and endorsement of specific AA-related behaviors and experiences, assessed 60 days post-baseline	Bivariate only	In the total sample, analyses showed marginally higher scores on the AAAS and a significantly higher rate of “sharing in meetings” among Black vs. White respondents; no other differences emerged.
Arroyo et al., 1998^65^	62 White and 46 Latinx respondents at baseline	Adults with AUD recruited from intake at the University of New Mexico’s outpatient, publicly funded SUD treatment program; longitudinal (follow-up rates 91% to 97%)	None described	Proportion days AA meeting attendance over the follow-up interval at 2, 4, and 6 months post-baseline	Multivariate only; controls were gender, education, and baseline AA attendance	In the total sample, analyses showed significantly fewer days of AA attendance among Latinx vs. White respondents collapsing across follow-ups.
Tonigan et al., 1998^66^	For outpatient sample, 735 White, 111 Latinx, and 52 Black respondents; for aftercare sample, 592 White, 27 Latinx, and 112 Black respondents at baseline	Project MATCH: Adults with AUD recruited from a broad range of SUD outpatient and residential treatment sites, assigned to one of three interventions; longitudinal (follow-up rates > 90%)	None described	AA attendance (yes vs. no) over the prior 3 months at 3, 6, 9, and 12 months post-baseline	Multivariate only; control was intervention condition	Among outpatients, analyses showed no racial/ethnic differences. Among aftercare patients, analyses showed significantly fewer days of AA attendance among both Latinx and Black vs. White respondents at the 12-month follow-up only; no other differences emerged.
Kaskutas et al., 1999^67^	538 White and 253 Black respondents at baseline	Epidemiological Laboratory (EpiLab) Study: Adults recruited from (a) 10 alcohol programs representative of public, HMO, and for-profit programs in northern California (*N* = 926) and (b) the general population of alcohol-dependent and problem drinkers (*N* = 672); analysis uses only sample (a); baseline analysis	N/A	AA and NA/CA attendance (yes vs. no); scores on a composite measure of AA involvement; and endorsement of specific AA-related behaviors/experiences, all for the pretreatment period and assessed at baseline	Bivariate and multivariate (the latter conducted only for AA attendance); controls were demographics, ASI Alcohol Severity, ASI Drug Severity, prior SUD treatment, and any prior NA/CA attendance	In the total sample, bivariate analyses showed significantly higher rates of both AA and NA/CA attendance among Black vs. White respondents. However, multivariate analyses showed no racial/ethnic differences in AA attendance. Among those reporting any AA attendance, there were no racial/ethnic differences in overall AA involvement, but significant differences emerged for specific AA-related behaviors/statuses: Black respondents were more likely to report that they were AA members, had had a spiritual awakening, and did service/volunteer work in the last year (vs. White respondents); White respondents were more likely to currently have a sponsor and to have read the AA literature (vs. Black respondents).
Hillhouse and Fiorentine, 2001^68^	76 White, 72 Latinx, and 110 Black respondents at follow-up	Adults (SUD status not specified) recruited from 26 outpatient SUD treatment programs in the Los Angeles area; only those in treatment for at least 8 weeks included; longitudinal (follow-up rate 74%)	None described	Pattern of 12-step participation (i.e., classification as persister, initiate, dropout, or nonattender) 24 months post-baseline	Bivariate only	In the total sample, analyses showed no racial/ethnic differences.
Tonigan, 2003^69^	1,380 White, 141 Latinx, and 168 Black respondents at baseline	Project MATCH: Adults with AUD recruited from a broad range of SUD outpatient and residential treatment sites, assigned to one of three interventions; baseline analysis	None described	Proportion days AA meeting attendance prior to treatment (period undefined), assessed at baseline	Bivariate only	In the total sample, analyses showed significantly fewer days of AA meeting attendance among both Latinx and Black vs. White respondents.
Goebert and Nishimura, 2011^70^	71 “Euro” American, 31 Asian American, and 90 Native Hawaiian respondents at baseline	Adults (SUD status not specified) recruited from intake at two major residential SUD treatment programs in Hawaii; baseline analysis	N/A	AA attendance (yes vs. no) prior to treatment (period undefined), assessed at baseline	Bivariate only	In the total sample, analyses showed no racial/ethnic differences.
Krentzman et al., 2012^71^	124 White and 41 Black respondents at baseline	Adolescents with SUD recruited from intake at the largest adolescent residential treatment provider in a central Midwestern region; longitudinal (follow-up rate 90%)	N/A	12-step helping behaviors and 12-step work in past month/90 days, as measured by Service to Others in Sobriety and General AA Tools of Recovery (GAATOR) scales, assessed 2 months post-baseline	Bivariate and multivariate; controls were baseline value of the outcome, demographics, total number of substance use diagnoses, prior SUD treatment, religiousness, readiness for change, and sexual abuse history	In the total sample, both bivariate and multivariate analyses showed no racial/ethnic differences.
Tonigan et al., 2013^72^	133 White and 63 American Indian respondents	Data merged from two studies recruiting adult participants in early AA affiliation and residing in large southwestern city; longitudinal (follow-up rates not specified)	N/A	Proportion days AA meeting attendance (period undefined), AA meeting attendance (yes vs. no), and 12-step work (assessed using GAATOR) at baseline and at 3, 6, and 9 months post-baseline	Multivariate only with time as the only covariate	In the total sample, analyses showed no racial/ethnic differences in AA attendance or 12-step work from baseline through follow-ups. However, analyses showed a significantly lower decline in any AA attendance over time among American Indian vs. White respondents.

*Note:* AA, Alcoholics Anonymous; AAAS, AA Affiliation Scale; ASI, Addiction Severity Index; AUD, alcohol use disorder; CA, Cocaine Anonymous; GAATOR, General AA Tools of Recovery; HMO, health maintenance organization; NA, Narcotics Anonymous; N/A, not applicable; SUD, substance use disorder.

* Analyses conducted on raw data by this review’s lead author.

**Table 3:** Summary of Results for Racial/Ethnic Disparities in Mutual Help Group Participation Across Studies

Comparison	Null Results	Mixed Results	Lower Minority Participation (Disparity)	Higher Minority Participation (Advantage)
Latinx vs. White	5 studies Table 1: Schmidt et al., 2007;^41^ Kaskutas et al., 2008;^60^ Perron et al., 2009;^61^ Wu et al., 2016;^62^ Table 2: Hillhouse and Fiorentine, 2001^68^	3 studies Table 1: Chartier et al., 2011;^38^ Mancini et al., 2015;^40^ Table 2: Tonigan et al., 1998^66^	4 studies Table 1: Cummings et al., 2011;^39^ Zemore et al., 2014;^42^ Table 2: Arroyo et al., 1998;^65^ Tonigan et al., 2003^69^	0 studies
Black vs. White	6 studies Table 1: Schmidt et al., 2007;^41^ Kaskutas et al., 2008;^60^ Wu et al., 2016;^62^ Table 2: Humphreys and Woods, 1993;^29^ Hillhouse and Fiorentine, 2001;^68^ Krentzman et al., 2012^71^	5 studies Table 1: Chartier et al., 2011;^38^ Zemore et al., 2014;^42^ Table 2: Kingree et al., 1997;^64^ Tonigan et al., 1998;^66^ Kaskutas et al., 1999^67^	2 studies Table 1: Cummings et al., 2011;^39^ Table 2: Tonigan et al., 2003^69^	2 studies Table 1: Perron et al., 2009;^61^ Table 2: Humphreys et al., 1991^63^
American Indian or Alaska Native vs. White	2 studies Table 1: Cummings et al., 2011;^39^ Table 2: Goebert and Nishimura, 2011^70^	1 study Table 2: Tonigan et al., 2013^72^	0 studies	1 study Table 1: Wu et al., 2016^62^
Asian American, Native Hawaiian or Other Pacific Islander vs. White*	2 studies Table 1: Cummings et al., 2011;^39^ Wu et al., 2016^62^	0 studies	0 studies	0 studies
Multiracial vs. White	2 studies Table 1: Cummings et al., 2011;^39^ Wu et al., 2016^62^	0 studies	0 studies	0 studies
Total Results	17 studies	9 studies	6 studies	3 studies

*Note:* Results coded as “mixed” when differing across outcomes, data sources, and/or subgroups (e.g., genders); marginally significant results coded as significant and not null.

* Comparisons were between Asian Americans vs. Whites and Native Hawaiians/Pacific Islanders vs. Whites^39^ and between Native Hawaiians/Pacific Islanders/Asian Americans vs. Whites.^62^
